# Comparative analysis of gut microbiota in healthy and diarrheic yaks

**DOI:** 10.1186/s12934-022-01836-y

**Published:** 2022-06-03

**Authors:** JunJun Liu, Xin Wang, Wenqian Zhang, Muhammad Fakhar-e-Alam Kulyar, Kalim Ullah, Zhaoqing Han, Jianhua Qin, Chongliang Bi, Yaping Wang, Kun Li

**Affiliations:** 1 College of Veterinary Medicine/Traditional Chinese Veterinary Medicine, Hebei Agriculture University, Baoding, 071001 People’s Republic of China; 2 College of Agriculture and Forestry, Linyi University, Shuangling Road, Linyi, Shandong 276005 People’s Republic of China; 3 Institute of Traditional Chinese Veterinary Medicine, College of Veterinary Medicine, Nanjing Agricultural University, Nanjing, 210095 People’s Republic of China; 4 MOE Joint International Research Laboratory of Animal Health and Food Safety, Nanjing Agricultural University, Nanjing, 210095 People’s Republic of China; 5 Department of Zoology, Kohat University of Science and Technology, Kohat, Khyber Pakhtunkhwa Pakistan; 6 College of Veterinary Medicine, Huazhong Agricultural University, Wuhan, 430070 China

**Keywords:** Yak, Diarrhea, Tibet Plateau, Gut microbiota

## Abstract

**Background:**

Yak (*Bos grunniens*) mainly inhabiting Tibet Plateau, displayed a high incidence of diarrhea due to harsh living environment and nutritional deficit. Gut microbial community has been reported to be closely related to many diseases including diabetes, obesity and inflammatory bowel disease, but information regarding diarrheic influence on gut microbiota in yaks remains scarce. Here, this study was performed to investigate the gut bacterial and fungal alternations of diarrheic yaks.

**Results:**

Results revealed that the gut bacterial and fungal communities of diarrheic yaks showed a distinct decline in alpha diversity, accompanied by significant shifts in taxonomic compositions. Specifically, diarrhea caused a distinct increase in the relative abundance of 1 phylum and 8 genera as well as a distinct decrease in 3 phyla and 30 genera. Fungal taxonomic analysis indicated that the relative richness of 1 phylum and 2 genera dramatically increased, whereas the relative richness of 2 phylum and 43 genera significantly decreased during diarrhea. Surprisingly, 2 bacterial genera and 5 fungal genera even cannot be detected in the gut microbiota of diarrheic yaks.

**Conclusions:**

In summary, this study indicated that the gut bacterial and fungal compositions and diversities of yaks altered significantly during diarrhea. Moreover, these findings also contribute to understanding the gut microbial composition and diversity of yaks and developing strategies to alleviate and prevent diarrhea from gut microbial perspective.

**Supplementary Information:**

The online version contains supplementary material available at 10.1186/s12934-022-01836-y.

## Introduction

Ruminant intestines harbor trillions of microbes, that serve key roles in metabolism, digestive absorption, intestinal homeostasis and host health [1, 2]. Additionally, gut microbiota has also been demonstrated to function in immune system maturation, permeability and epithelial differentiation of intestine [3, 4]. Gut-residing beneficial bacteria can limit the colonization of foreign pathogens in the intestine via secreting antimicrobial peptide, regulating intestinal environment and competing nutrition, which was deemed as a natural barrier against pathogenic bacteria invasion [5, 6]. Statistically, the normal intestine contains more than 10^14^ microbes, approximately 10 times the total quantity of host cells [7]. Among them, intestinal bacteria account for approximately 98% of the total microbial community, whereas the rest contains fungi (0.1%), viruses and protozoa [7–9]. The stabilized gut microbiota is required for various complex physiological and metabolic processes, whereas gut microbial dysbiosis may result in multiple gastrointestinal diseases including diarrhea, enteritis and irritable bowel syndrome [10, 11]. Although these microbes colonize the intestine, they may result in systemic effects [12]. Increasing evidence suggests that gut microbial dysbiosis has an impact on intestinal functions with negative influence beyond the gastrointestinal system, impairing the functioning of other organs including liver and brain [13, 14].

Diarrhea is a common disease that is often accompanied by gastrointestinal dysfunction and may cause decreased productivity, weakened immunity and even mortality [15, 16]. Numerous studies demonstrated that diarrhea occurred in almost all species, especially in newborn piglets, goats and cattle with immature gut microbiota, which was regarded as one of the important constraint to livestock sector [17, 18]. Certain gut-residing bacteria and fungi change between dominant and weak populations accompanied by diarrhea, indicating some inevitable relationships may exist between gut microbial dysbiosis and diarrhea, but specific links and laws are still unclear [19, 20].

Currently, high-throughput sequencing technology has been successfully applied to investigate gut microbial alterations after the onset of various diseases, making it possible to deeply characterize the potential relationship between gut microbiota and some diseases [6, 21]. Furthermore, in-depth analysis of the complicated gut microbiota is beneficial to further understand the mechanisms contributing to ill health and timely formulate strategies to minimize the collateral damage [22]. Yak is an aboriginal breed mainly inhabits in the Tibet Plateau (average elevation above 4000 m), characterized by cold resistance and strong adaptability [23, 24]. This indigenous breed has resided in the high-altitude hypoxic environment for thousands of years and has evolved unique digestive characteristics and gut microbiota that contribute to adapting to high-fiber diet, but also make them susceptible to various gastrointestinal diseases, especially diarrhea [25, 26]. Consequently, the gut microbiota of yaks plays a more noticeable role in various physiological functions compared with poultry and other mammal. However, the potential relationship between the gut microbiota in yaks and diarrhea remain to be determined. In this study, we investigated the gut bacterial and fungal compositions and variabilities of healthy and diarrheic yaks.

## Materials and methods

### Sample collection

In the present study, a total of 12 12-month-old free-range yaks with similar blood profile and weight (6 healthy and 6 diarrheic) were selected for sample collection from Qinghai Province, China, including 3 male and female yaks in each group. The diarrheic yaks were diagnosed by professional veterinarian and didn’t received any treatment before sample acquisition. On the day of sample acquisition, each yak was placed in a separate enclosure to prevent potential contamination between samples. The rectal feces (approximately 200 g) were collected from each selected yak using sampler. The obtained fresh fecal samples were sub-sampled from the central proportion to decrease contamination via flooring and bedding. The final samples were snap-frozen using liquid nitrogen and stored at − 80 ℃ for further analysis.

### DNA extraction and high-throughput sequencing

Total bacterial and fungal genomic DNA were extracted from 12 frozen feces (approximately 200 mg) of healthy and diarrheic yaks utilizing QIAamp DNA Mini Kit (QIAGEN, Hilden, Germany) based on manufacturer’s protocol recommendations. To further ensure the quality of extracted total genomic DNA, 0.8% agarose gel electrophoresis and UV–Vis spectrophotometer (NanoDrop 2000, United States) were used for evaluating the integrity and concentration of the extract, respectively. The universal primers including bacterial 16S rDNA (338F: ACTCCTACGGGAGGCAGCA and 806R: GGACTACHVGGGTWTCTAAT) and fungal ITS (ITS5F: GGAAG TAAAAGTCGTAACAAGG and ITS2R: GCTGCGTTCTTCATCGA TGC) gene primers were used for amplifying V3/V4 hypervariable and ITS2 regions, respectively. Subsequently, the amplified products were evaluated by 2.0% agarose gel electrophoresis and then subjected for purification and recycle. The recycled product were conducted fluorescent quantitation and the samples were mixed on a pro-rata basis following the fluorescence quantitative result and sequencing amount requirement. Based on the manufacturer’s protocol, the purified products were applied to construct sequencing library utilizing Illumina TruSeq (Illumina, United States). Prior to the sequencing, the prepared libraries required to be further processed including purification, quality control and fluorescence quantification. The libraries that passed the quality inspection and showed only one peak were considered qualified. Finally, the qualified libraries were diluted and denatured to single-stranded and then performed 2 × 300 bp paired-end sequencing.

### Bioinformatics and statistical analysis

The raw data need to be preprocessed to obtain reliable results for subsequent analysis. The raw reads were performed quality filtering using Trimmomatic software (v0.33). Afterwards, the Cutadapt software (1.9.1) was employed to recognize and remove primer sequences to obtain clean reads. The clean reads were spliced using Usearch software (v10) and the sequences less than 200 bp were abandoned. Additionally, the Uchime software (v4.2) was used for identifying and eliminating chimera to obtain the final effective reads. The effective reads were obtained through clustering and OTU discrimination based on 97% nucleotide-sequence similarity. The Ribosome Database Program classifier and MUSCLE software were used for recognizing the representative sequence and performing phylogenetic analysis of each OTU, respectively. Prior to conducting the bacterial and fungal diversities analysis, the rank abundance and rarefaction curve were structured to dissect sequencing depth. The gut bacterial and fungal diversities indices including Good's coverage, ACE, Simpson and Shannon were generated based on the relative abundance of OTU in each sample. Additionally, beta diversity analysis was also conducted to dissect the differences of the main components in different samples. LEfSe was used for identifying the taxa related to diarrhea in gut bacterial and fungal communities. Statistical analysis was conducted to assess the differences of both groups using GraphPad Prism (version 8.0c) and SPSS statistical program (v19.0). Probability values (means ± SD) < 0.05 were considered statistically significant.

## Results

### Sequence analysis

**Fig. 1:**
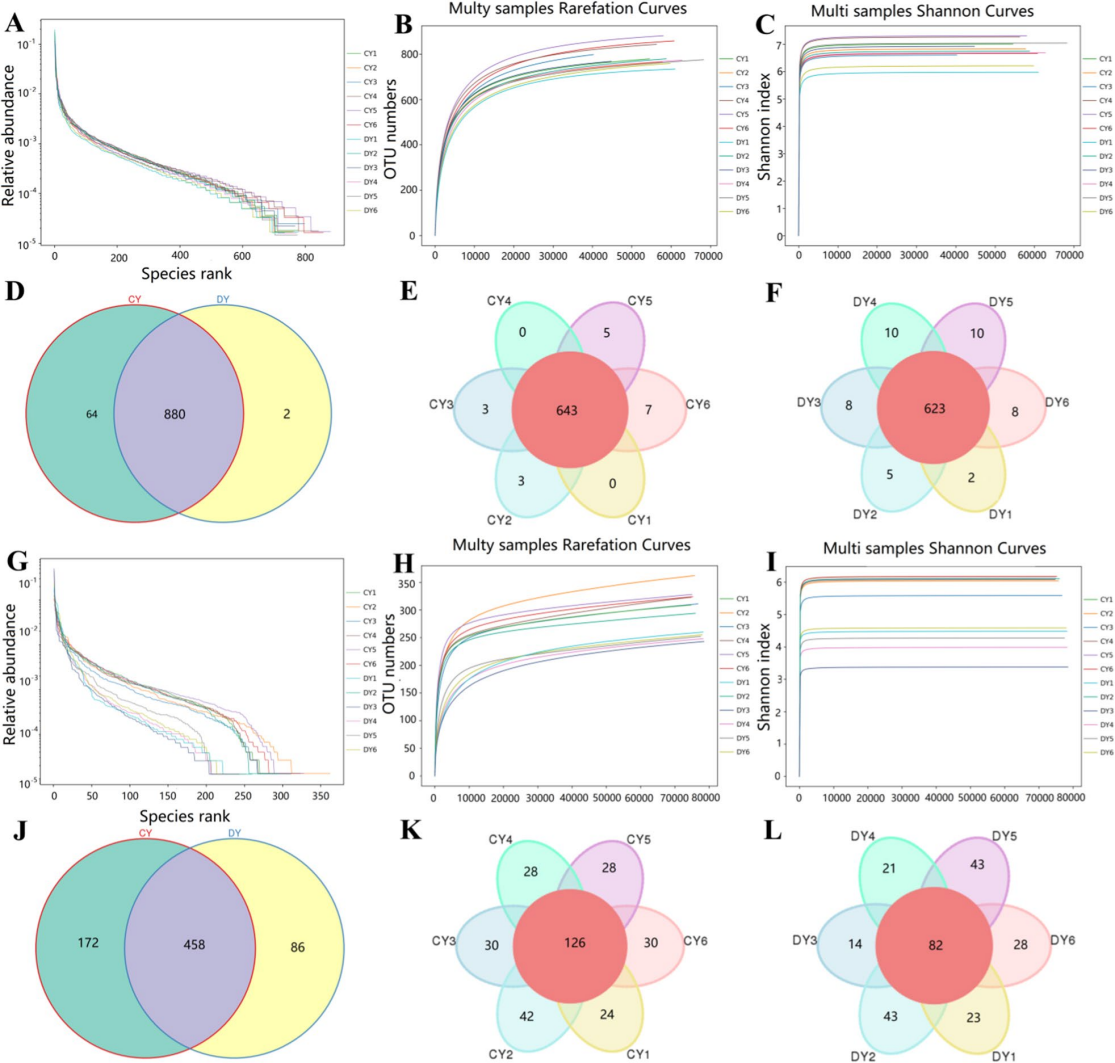
Sequencing data feasibility analysis and OTUs distribution. **A**, **B** Bacterial rarefaction curves for all samples. **C** Bacterial Rank-Abundance curve. **D**–**F** Gut bacterial OTUs distribution in different samples. **G**, **H** Fungal rarefaction curves for all samples. **I** Bacterial Rank-Abundance curve. **J**–**L** Gut fungal OTUs distribution in different samples

To investigate the gut microbial alterations of yaks during diarrhea, 12 (6 control samples and 6 diarrheic samples) fecal samples were subjected to amplicon sequencing. We totally acquired 907,882 (CY = 451,996, DY = 455,886) and 959,714 (CY = 479,401, DY = 480,313) raw sequences from the V3/4 and ITS2 regions of collected samples, respectively (Table 1). After quality evaluation, a total of 1813,360 (CY = 863,743, DY = 949,617) high-quality sequences were achieved, with a median read count of 71,978 (varying from 49,427 to 77,974) and 79,134 (ranging from 78,367 to 79,540) reads from bacterial and fungal communities per sample, respectively (Table 2). The rarefaction curves and shannon curves of each sample was invariably extended to the right end of the x-axis and showed a saturated tendency, indicating that the quantity and depth of sequencing met the demands of further analysis (Fig. 1A–C, G–I). Following taxonomic assignment, the sequences generated from the V3/4 and ITS2 regions were clustered into 946 bacterial OTUs and 716 fungal OTUs on the basis of 97% sequence similarity (Fig. 1E, F, K, L). Furthermore, 880 OTUs were shared in the bacterial community as well as 458 core OTUs were recognized in the fungal community, accounting for approximately 93.02% and 63.97% of the overall OTUs, respectively (Fig. 1D, J).

**Table 1:** The bacterial sequence information of each sample

Sample	Raw reads	Clean reads	Effective reads	AvgLen (bp)	GC (%)	Effective (%)
CY1	79,685	79,254	75,775	413	52.98	95.09
CY2	79,659	79,225	75,510	411	53.9	94.79
CY3	52,648	52,283	49,427	415	53.36	93.88
CY4	79,852	79,428	75,427	413	53.15	94.46
CY5	79,931	79,520	76,049	413	53.22	95.14
CY6	80,221	79,760	76,094	411	53.92	94.86
DY1	79,904	79,370	75,301	417	53.14	94.24
DY2	79,790	79,383	76,570	412	53.32	95.96
DY3	56,330	55,886	53,836	414	53.37	95.57
DY4	79,792	79,317	76,180	415	53.15	95.47
DY5	80,074	79,621	77,974	412	53.29	97.38
DY6	79,996	79,535	75,600	416	53.11	94.50

**Table 2:** The fungal sequence information of each sample

Sample	Raw reads	Clean reads	Effective reads	AvgLen (bp)	GC (%)	Effective (%)
CY1	79,885	79,360	78,367	252	45.14	98.10
CY2	79,829	79,227	79,114	255	45.22	99.10
CY3	79,926	79,358	79,188	250	45.13	99.08
CY4	80,077	79,455	78,702	252	44.66	98.28
CY5	79,762	79,070	78,772	251	44.45	98.76
CY6	79,922	79,432	79,271	250	44.37	99.19
DY1	79,852	79,323	79,260	250	46.09	99.26
DY2	80,084	79,471	79,405	254	45.37	99.15
DY3	80,187	79,775	79,455	240	46.21	99.09
DY4	80,229	79,725	79,540	238	46.37	99.14
DY5	79,875	79,276	79,016	239	46.04	98.92
DY6	80,086	79,602	79,527	255	45.53	99.30

### Bacterial and fungal diversities in fecal microbiota associated with diarrhea

**Fig. 2:**
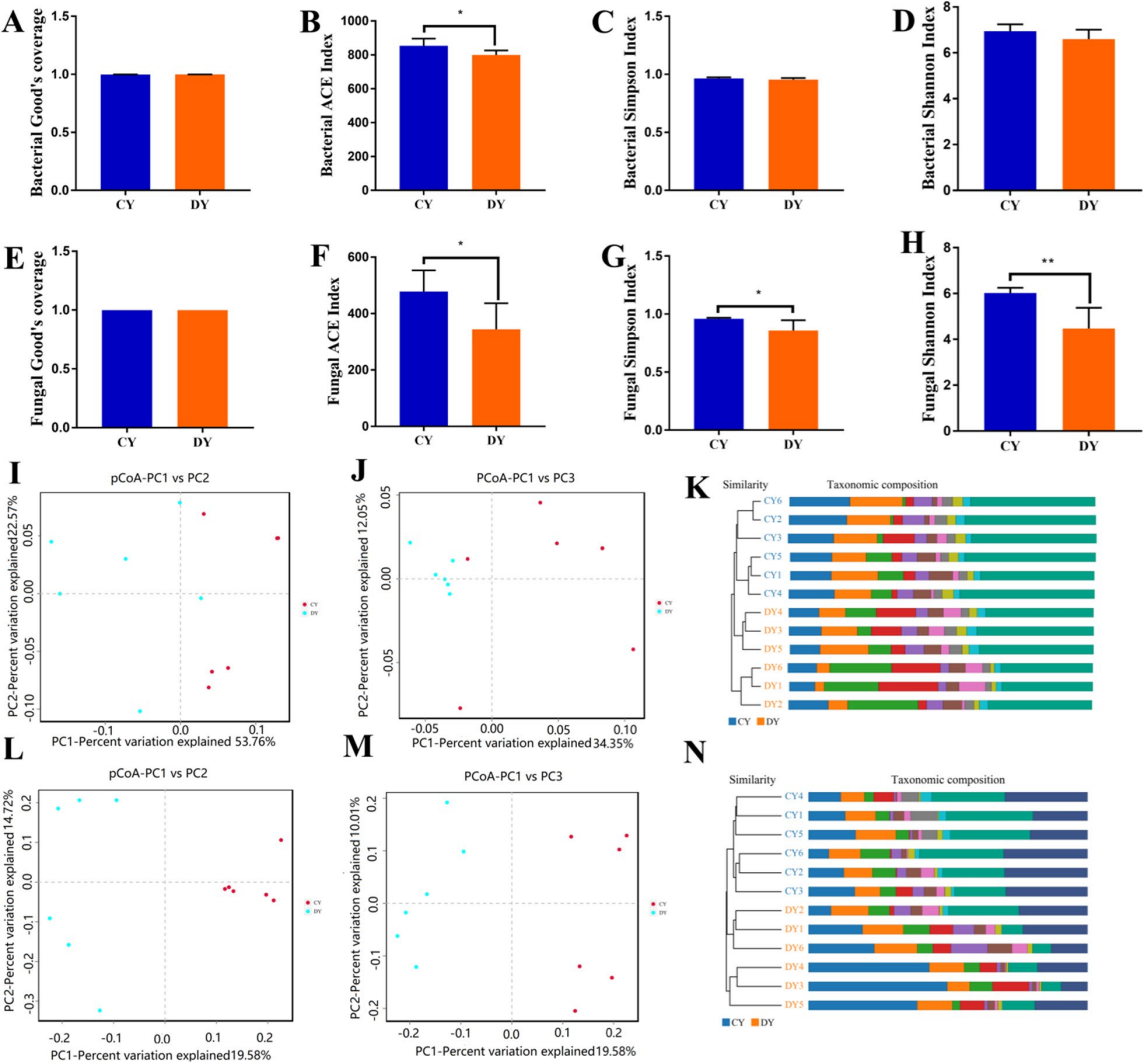
Comparative analysis of the gut bacterial and fungal diversities between healthy and diarrheic yaks. **A**, **E** Good’s coverage index. **B**, **F** ACE index. **C**, **G** Simpson index. **D**, **H** Shannon index. **I** Weighted UniFrac PCoA plots. **J** Unweighted UniFrac PCoA plots. PCoA plots revealed the gut fungal main component differences between both groups (**L**, **M**). **K** Gut bacterial clustering analysis. **N** Gut fungal clustering analysis. All of the data represent means ± SD. *p < 0.05, **p < 0.01

To further characterize the shifts of gut microbial community in yaks during diarrhea, we calculated the multiple alpha and beta diversity indices of the gut microbiota. Results demonstrated that the Good's coverage estimates in the bacterial and fungal populations ranged from 99.79% to 99.93% and 99.93% to 99.95%, respectively, implying that the most of bacterial and fungal phenotypes in the samples could be detected (Fig. 2A, E). Moreover, statistical analysis revealed that the bacterial ACE index (854.09 17.22 versus 799.86 10.72, p = 0.032) differed significantly, but the Simpson (0.96 ± 0.0041 versus 0.95 ± 0.0059, p = 0.209) and Shannon (6.9466 ± 0.12 versus 6.59 ± 0.17, p = 0.13) indices were not significantly different between CY and DY groups (Fig. 2B–D). These results demonstrated that the gut bacterial diversity yaks was strongly influenced by the diarrhea. Additionally, we also observed a obvious decrease in the gut fungal diversity index during diarrhea including ACE, Simpson and Shannon, suggesting that diarrhea observably reduce the gut fungal diversity and abundance of yaks (Fig. 2F–H). The principal coordinate analysis (PCoA) was used for evaluating the similarity and variability between intergroup and intra-group samples. PCoA scatterplot of gut bacterial and fungal communities revealed a separation of individuals in the CY and DY groups, which was in line with the UPGMA analytical results, suggesting that the gut microbial principal compositions of yaks were strongly influenced by the diarrhea (Fig. 2I–N).

### Significant alterations of bacterial taxonomic composition in diarrheic yaks

**Fig. 3:**
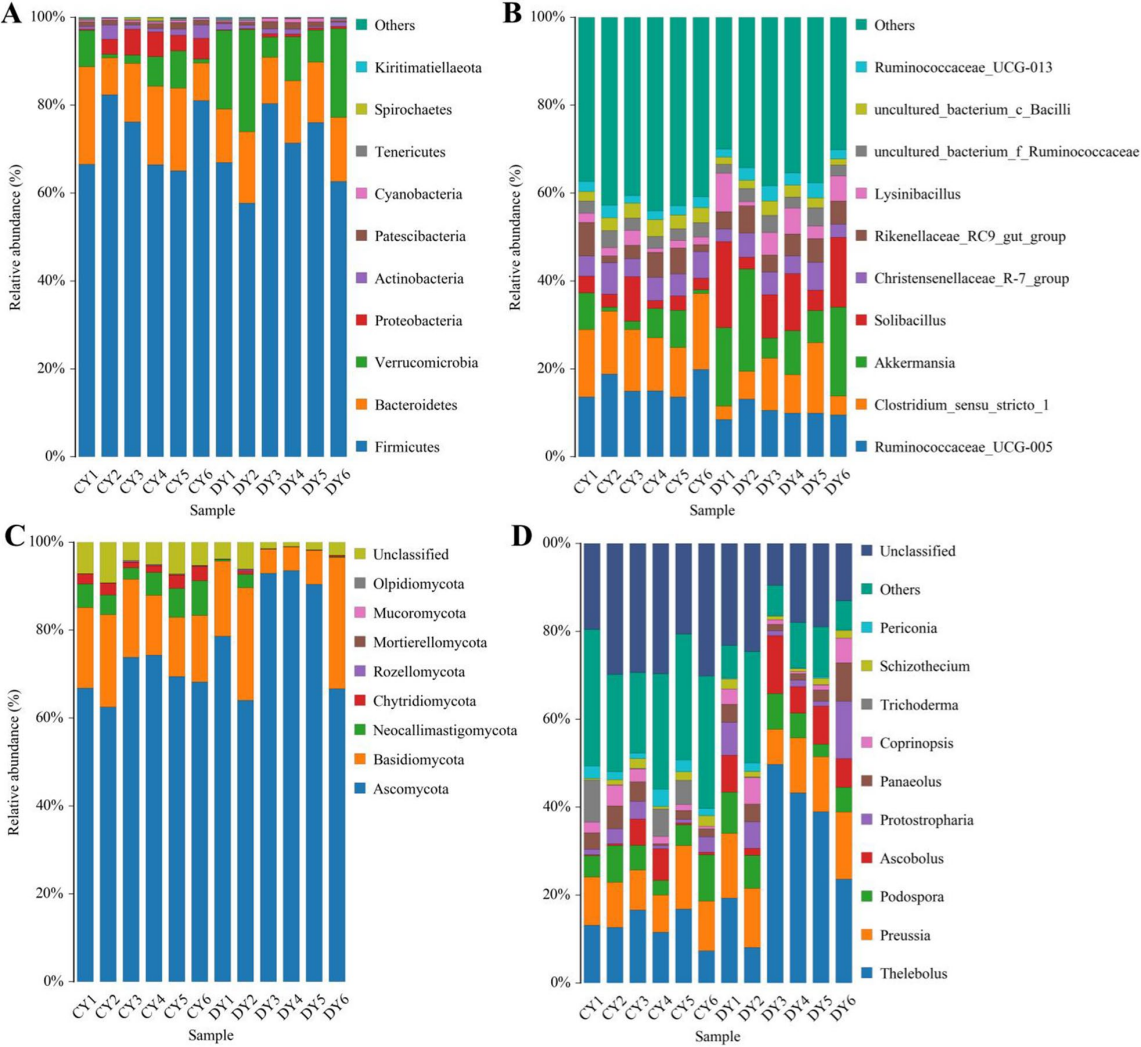
The relative abundances and distribution of preponderant bacteria and fungi in healthy and diarrheic yaks. Gut bacterial composition at the phylum (**A**) and genus (**B**) levels. Gut fungal composition at the phylum (**C**) and genus (**D**) levels. Clustered heatmap of yaks in different health status at the genus level. The color values of the heatmap indicate the normalized relative richness of each species

The relative abundances of preponderant taxa at the phylum and genus level were determined using microbial taxon assignment and distinct shifts were observed in the taxonomic composition between both groups during diarrhea. At the phylum level, 16 phyla were recognized in the gut bacterial community, ranging from 13 to 16 phyla per sample. The phyla *Firmicutes* (72.95%), *Bacteroidetes* (14.80%) and *Verrucomicrobia* (4.58%) were the three most dominant phyla in CY group, which together consisted of total 92.33% of the bacterial composition (Fig. 3A). Moreover, *Firmicutes* (68.95%) was the most preponderant bacterial phylum in the DY groups, followed by the *Bacteroidetes* (13.67%) and *Verrucomicrobia* (14.09%), accounting for approximately 96.71% of all bacterial taxa. Other phyla such as *Cyanobacteria* (CY = 0.39%, DY = 0.56%), *Tenericutes* (CY = 0.24%, DY = 0.26%), *Spirochaetes* (CY = 0.18%, DY = 0.035%) and *Kiritimatiellaeota* (CY = 0.089%, DY = 0.10%) in CY and DY groups were detected in lower abundances. To further evaluate the shifts of gut bacterial compositions during diarrhea, a total of 191 genera were recognized. Among them, *Ruminococcaceae_UCG-005* (16.11%) was the most dominant genus in the CY group, followed by *Clostridium_sensu_stricto_1* (14.08%) and *Christensenellaceae_R-7_group* (5.46%) (Fig. 3B). Meanwhile, *Akkermansia* (14.09%), *Solibacillus* (10.92%) and *Ruminococcaceae_UCG-005* (10.23%) were abundantly present in the DY group, which accounted for approximately 35.24% of the total taxonomic groups identified. The clustering heatmap also revealed the distribution of the identified bacterial genus as well as a variability of the gut bacterial community in diarrheic yaks (Additional file 1: Fig. S1).

**Fig. 4:**
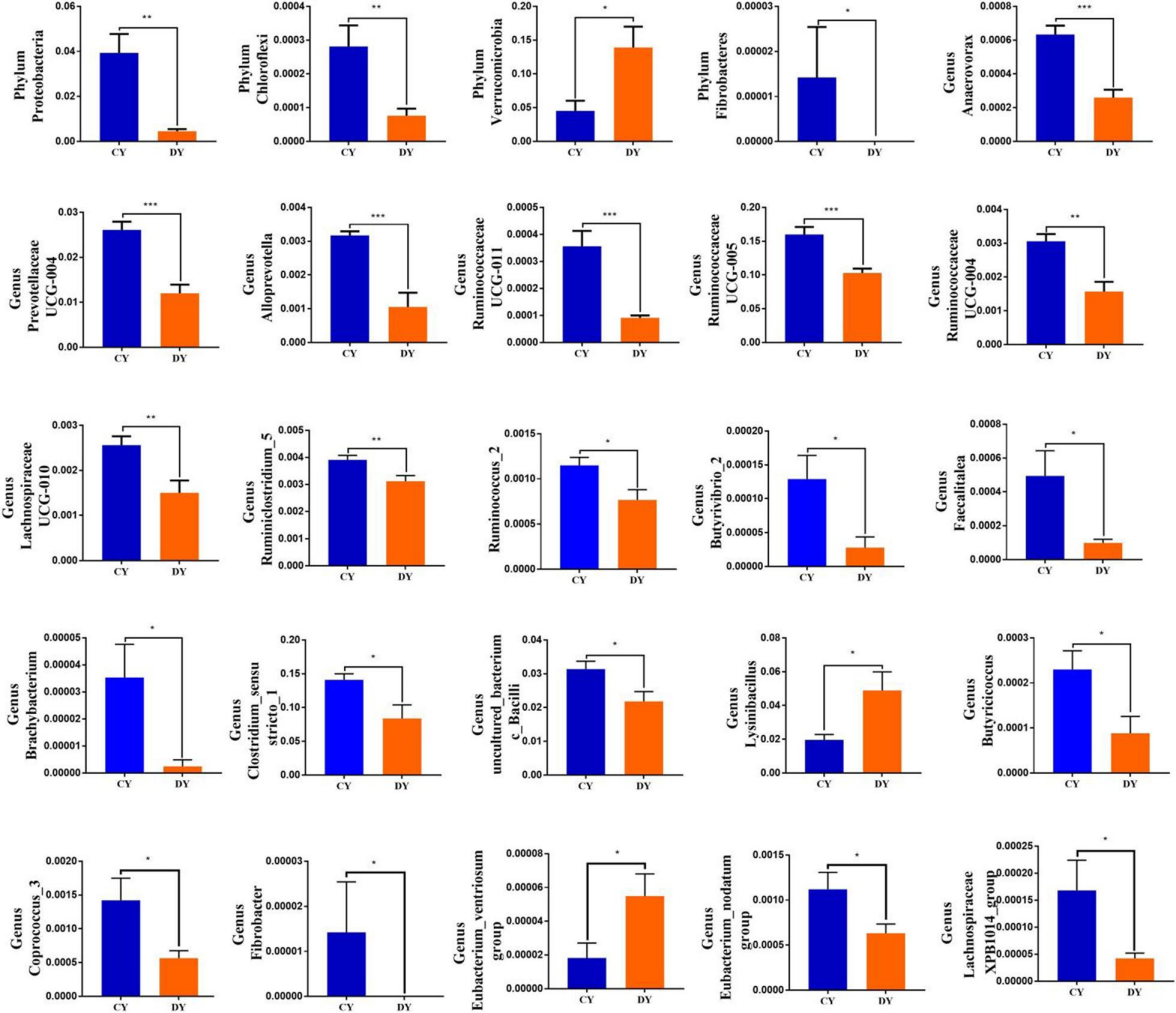
The gut bacterial comparisons between healthy and diarrheic yaks in phylum and genus levels. Metastats analysis was applied to identify the significantly differentially abundant bacterial genera between both groups and all of the data represent means ± SD. *p < 0.05, **p < 0.01

To further characterize the changes of taxonomic composition in yaks during diarrhea, Metastats analysis was conducted for different classification levels (Fig. 4 and Additional file 1:Fig. S3). At the phylum level, *Proteobacteria*, *Chloroflexi* and *Fibrobacteres* were dramatically more preponderant in the CY group than in the DY group, whereas the *Verrucomicrobia* was lower (P < 0.05 or P < 0.01). Moreover, a comparison of the DY and CY groups indicated a distinct decline in the richness of 30 bacterial genus as well as a significant increase in the richness of 8 bacterial genus. Given that this discriminant analysis may not detect the whole taxa, LEfSe coupled with LDA scores was applied to recognize the specific bacteria associated with diarrhea (Fig. 6A, B). Besides the above-mentioned differential taxa, we also found that several bacterial genera including *uncultured_bacterium_f_Planococcaceae* was the most preponderant in the DY group, whereas *Escherichia_Shigella* was dramatically overrepresented in the CY group.

### Significant shifts of gut fungal compositions in yaks during diarrhea

In this study, a total of 8 phyla and 248 genera were identified in gut fungal community and the main preponderant phyla and genera were shown in Fig. 3C, D. Specifically, the phyla *Ascomycota* (CY = 69.20%, DY = 81.14), *Basidiomycota* (CY = 16.57%, DY = 15.15%) and *Neocallimastigomycota* (CY = 5.34%, DY = 0.60%) were abundantly present in both groups, regardless of health status, accounting for over 90% of total fungi taxa. Other fungal phyla including *Rozellomycota* (CY = 0.050%, DY = 0.065%), *Mortierellomycota* (CY = 0.075%, DY = 0.035%), *Mucoromycota* (CY = 0.083%, DY = 0.014%) and *Olpidiomycota* (CY = 0.014%, DY = 0.0011%) in CY and DY groups were identified in a low richness. Among identified genera, *Thelebolus* (13.04%) was the most dominant fungal genera in the CY group, followed by *Preussia* (10.75%) and *Podospora* (6.26%). However, the preponderant fungal genera recognized in the DY group were *Thelebolus* (30.62%), *Preussia* (12.74%) and *Ascobolus* (7.43%), which were different from the CY group. The distribution and relative richness of identified fungal genera were further investigated by clustering analysis indicated by the heatmap (Additional file 1: Fig. S2).

**Fig. 5:**
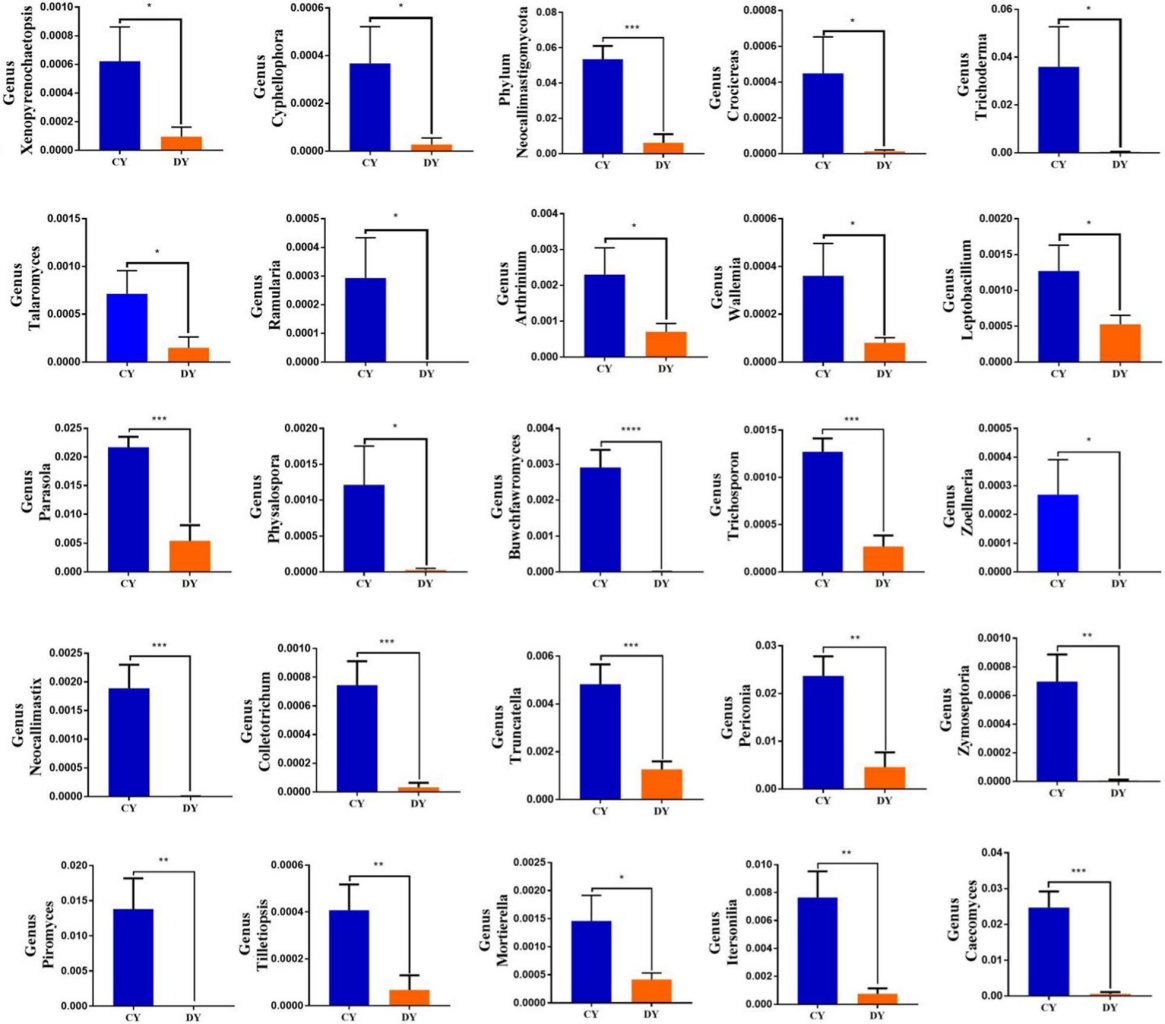
The gut fungal comparisons between healthy and diarrheic yaks in phylum and genus levels. Metastats analysis was applied to identify the significantly differentially abundant fungal genera between both groups and all of the data represent means ± SD. *p < 0.05, **p < 0.01

**Fig. 6:**
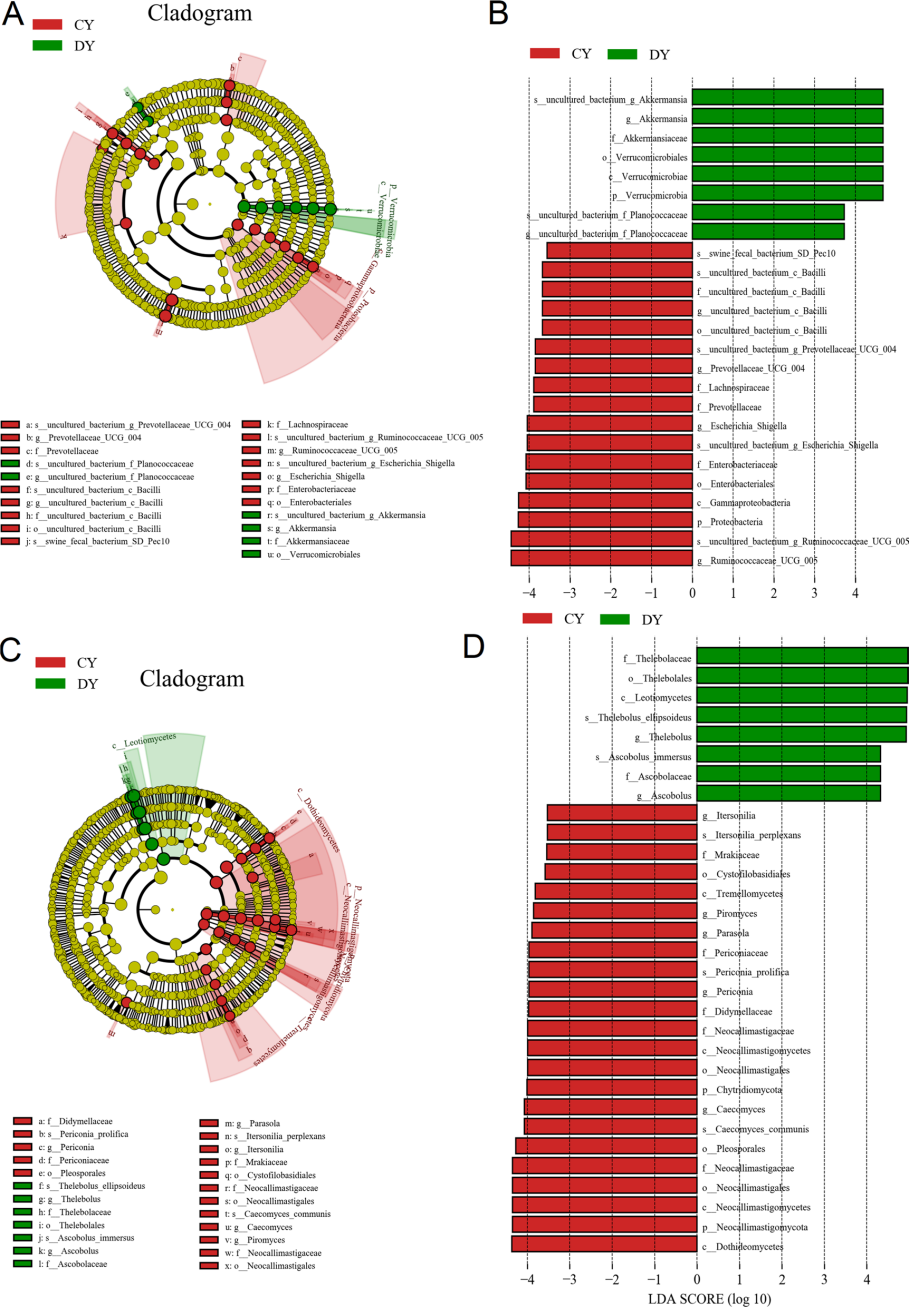
Integrated LEfSe analysis and LDA scores displayed the differential taxa related to diarrhea. **A**, **C** Cladogram demonstrating the phylogenetic distribution of bacteria and fungi related to diarrhea. **B**, **D** The criterion of significance was performed at LDA scores > 3.5

Using Metastats analysis to compare the differences in the gut fungal community of both groups (Fig. 5 and Additional file 1: Fig. S4). At the phylum level, *Chytridiomycota* and *Neocallimastigomycota* in DY group were significantly reduced, whereas *Ascomycota* was significantly increased as compared to CY group (p < 0.05 or p < 0.01). Additionally, 45 fungal genera were detected to be significantly different between CY and DY groups. Of these differential taxa, the relative richness of 43 fungal genera distinctly decreased, while 2 fungal genera obviously increased in diarrheic yaks. Among decreased fungus, 4 genera including *Humicola*, *Mucor*, *Ramularia* and *Zoellneria* even could not be detected in the gut fungal community. LEfSe combined with LDA scores were used for further dissecting the alternations of gut fungal composition (Fig. 6C, D).

### Correlation network analysis

**Fig. 7:**
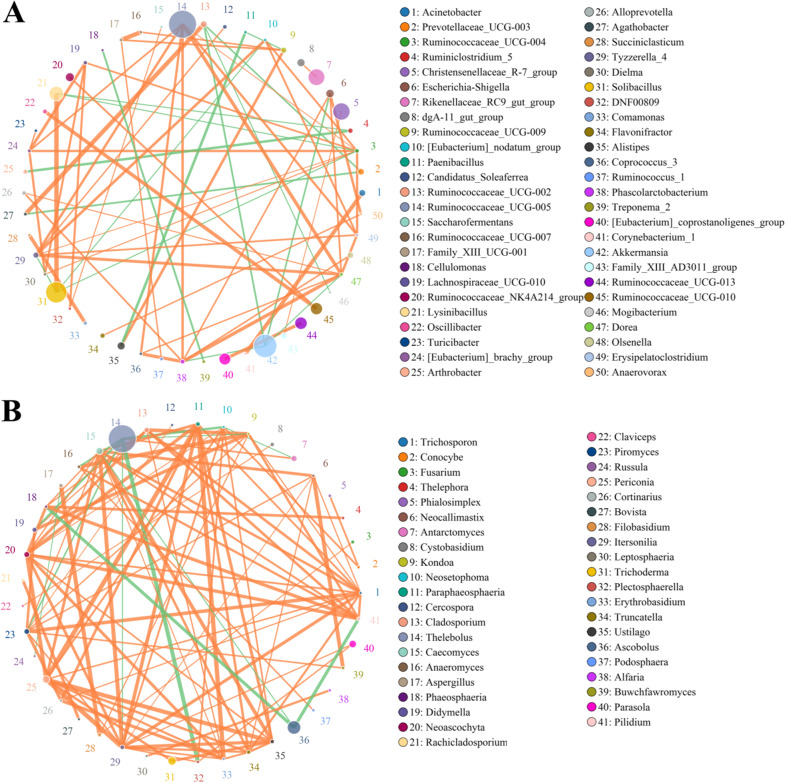
Network analysis indicates the connection between different bacteria and fungi. The different node color represents various bacterial and fungal taxa and the weighted node size was determined according to the relative abundance. The orange lines indicate positive correlation, while green lines indicate negative correlation

Network analysis was performed to dissect the correlations between different bacteria or fungi of gut microbial community (Fig. 7). Results demonstrated that *Dorea* was positively associated with *Coprococcus_3* (0.8531) and *Erysipelatoclostridium* (0.8951). *Caecomyces* was positively correlated with *Neoascochyta* (0.8112), *Piromyces* (0.8042), *Paraphaeosphaeria* (0.8671), *Itersonilia* (0.9231), *Erythrobasidium* (0.7972), *Paraphaeosphaeria* (0.8671), *Phaeosphaeria* (0.8112) and *Trichosporon* (0.8601). *Ruminococcaceae_UCG-004* was positively associated with *Lachnospiraceae_UCG-010* (0.8671) and *Erysipelatoclostridium* (0.8881). *Ruminococcaceae_UCG-005* was positively associated with *Tyzzerella_4 *(0.8951), *Coprococcus_3 (0.8881)*, *Erysipelatoclostridium* (0.8881), *Dorea* (0.9301) and *Ruminococcaceae_UCG-004* (0.8322). *Parasola* was positively correlated with *Itersonilia* (0.8042) and *Plectosphaerella* (0.8112). *Neoascochyta* was positively related to *Piromyces* (0.7273), *Itersonilia* (0.8741), *Truncatella* (0.7902) and *Buwchfawromyces* (0.5873). *Truncatella* was positively related to *Erythrobasidium* (0.8322), *Neocallimastix* (0.8049) and *Trichosporon* (0.8462). *Periconia* was positively related to *Didymella* (0.6434), *Caecomyces* (0.8741), *Paraphaeosphaeria* (0.9441), *Neosetophoma* (0.8371), *Trichosporon* (0.8182), *Ustilago* (0.7273), *Truncatella* (0.6783), *Erythrobasidium* (0.8462) and *Itersonilia* (0.9441). *Itersonilia* was positively associated with *Neosetophoma* (0.8336), *Neocallimastix* (0.7904), *Plectosphaerella* (0.8182), *Erythrobasidium* (0.8252) and *Ustilago* (0.8881). *Piromyces* was positively correlated with *Didymella* (0.8601), *Cortinarius* (0.8811), *Itersonilia* (0.8182), *Buwchfawromyces* (0.8194), *Trichosporon* (0.9021), *Neocallimastix* (0.8121), *Kondoa* (0.8112) and *Cercospora* (0.7902). *Caecomyces* was positively related to *Phaeosphaeria* (0.8112), *Neoascochyta* (0.8112), *Itersonilia* (0.9231), *Erythrobasidium* (0.7972), *Ustilago* (0.9091), *Pilidium* (0.8392), *Trichosporon* (0.8601), *Neocallimastix* (0.8774) and *Paraphaeosphaeria* (0.8671). *Pilidium* was positively associated with *Phaeosphaeria* (0.8951), *Anaeromyces* (0.8531) and *Kondoa* (0.8601). *Paraphaeosphaeria* was positively related to *Cortinarius* (0.8671), *Itersonilia* (0.9021), *Erythrobasidium* (0.9371), *Truncatella* (0.8601) and *Trichosporon* (0.8322).

## Discussion

Diarrhea is one of the most prevalent diseases of farm animals regardless of species and age, which was regarded as a vital factor causing the decreased global animal productivity [20]. The yaks mainly inhabiting the Tibet plateau possess a high rate of diarrhea, causing substantial economic loss to the yak industry [27]. However, multiple factors including hostile environment, nutritional imbalance and stress response cause the control of diarrhea in yaks particularly difficult [28]. Gut microbial significance has been extensively acknowledged due to its positive roles in immunity, metabolism and intestinal barrier [3, 4]. Moreover, recent studies on gut microbiota have revealed its important role in the control of diarrhea [5, 29]. Therefore, the high diarrhea rate of the yaks may not only be related to their habitat environment but also involve gut microbiota. Systematically understanding the gut microbial information is beneficial to further investigate the disease etiology and develop the potential treatment and prevention options to minimize collateral damage [30]. Currently, research into the gut microbial community of diarrheic ruminant has covered many aspects including goat, cattle and giraffe, but knowledge regarding the gut microbiota in diarrheic yaks remains scarce [16, 18]. In this study, we deeply analyzed and compared the differences of gut microbiota between healthy and diarrheic yaks and revealed a high variability of gut microbial composition and diversity of diarrheic yaks.

Anecdotal evidence indicated that the gut microbiota vary dynamically within limits and affected by species, age and feed, but these physiological fluctuations cannot impair the normal intestinal functions [31, 32]. However, the ecological balance of of gut microbiota can be disrupted by multiple factors including diabetes, antibiotics and diarrhea [33, 34]. Wang et al. reported that the gut bacterial diversity of diarrheic Boer goats decreased dramatically as compared with healthy populations [16]. Moreover, Li et al. also indicated a decreased gut bacterial diversity of giraffes during diarrhea [35]. Consistent with previous investigations, the present study revealed a significantly decreased gut bacterial diversity of diarrheic yaks, suggesting the gut bacterial dysbiosis. The intestine is the leading absorption site, which depends on the normal gut microbial composition and diversity [36]. Early investigations have indicated that the normal gut microbial composition and diversity were the prerequisite for performing its complex physiological functions, while gut microbial dysbiosis may be the central or driving factor of multiple diseases [37, 38]. Previous research demonstrated that the gut microbial dysbiosis might be one of the reasons for the high mortality of diarrheic goats [16]. Additionally, diarrheic yaks are characterized by high mortality and weight loss. Several previous studies indicated that gut microbial dysbiosis can affect host immunity and intestinal permeability, thereby increasing the susceptibility to pathogens [36, 39]. Moreover, some conditioned pathogen may also display pathogenicity under circumstances of gut microbial dysbiosis [36]. Therefore, we speculated that the gut microbial dysbiosis may be one of the reasons for the high mortality and weight loss of diarrheic yaks. As a part of gut microbiota, the intestinal fungus is considered as a vital participant in intestinal functions and host health [40]. Similarly, we also observed a obvious reduction in the gut fungal diversity in diarrheic yaks, indicating the gut fungal dysbiosis. Furthermore, beta diversity analysis showed that despite sharing the same habitat and diet, the major components of healthy and diarrheic yaks' gut bacterial and fungal communities were substantially different, demonstrating that the diarrhea may be a fundamental driving factor for shifts in gut bacterial and fungal communities.

This study indicated that *Firmicutes*, *Bacteroidetes*, *Ascomycota* and *Basidiomycota* were the most preponderant microbial phyla in yaks, regardless of health status, which was in line with previous investigations on yaks [24]. Additionally, these dominant phyla were also demonstrated to be extensively existed in the goats, giraffes and cattle, indicating their importance in intestinal ecology and functions in ruminants [31, 41]. Some specific bacterial and fungal alternations may reflect the potential relationship between diarrhea and gut microbiota, thus we further characterized the intestinal bacteria and fungi associated with diarrhea. Results indicated a significant increase in 1 bacterial phylum (*Verrucomicrobia*) and 1 fungal phylum (*Ascomycota*) as well as a decrease in 3 bacterial phyla (*Proteobacteria*, *Chloroflexi* and *Fibrobacteres*) and 2 fungal phyla (*Chytridiomycota* and *Neocallimastigomycota*) in diarrheic yaks. The phylum *Proteobacteria* has been demonstrated to be abundantly present in the gastrointestinal tract of yaks and contribute to meeting their high nutrient and energy requirements, due to the highly diverse metabolic functions [24, 42]. Moreover, anecdotal evidence indicated that the most members of phylum *Fibrobacteres* can degrade cellulose [43]. Consistent with the present observations, Wang et al. also revealed that the relative abundance of *Verrucomicrobia* in diarrheic goats was dramatically increased [16]. Most members of phylum *Chytridiomycota* can decompose cellulose and chitin. Similarly, the members of *Neocallimastigomycota* mainly inhabit the digestive tract of mammals, which have the ability to decompose cellulose [44, 45]. Diarrhea is a common gastrointestinal diseases in yaks, which is inevitably accompanied by weight loss. As a strict herbivorous ruminant, the yaks need to consume a large amount of forage to maintain their energy consumption and growth on the high-altitude hypoxic environment of Tibetan plateau [46]. However, we observed that some bacteria associated with cellulose degradation decreased significantly during diarrhea, indicating a decreased ability to digest and degrade food. We presumed that this may be one of the important reasons for the weight loss of yaks during diarrhea.

Importantly, we also found considerable variability in some bacterial and fungal taxonomic taxa of diarrheic yaks, which may play vital roles in gut microbial balance and intestinal functions. Interestingly, most of the altered bacterial phyla and genera in diarrhea yaks showed a downward trend and even 2 bacterial genera could not be detected, implying that these bacteria cannot adapt to the current intestinal environment. We speculated that the intestinal environment of the diarrheic yaks may undergo significant alternations, which in turn selectively inhibited the colonization of some bacteria. Additionally, some decreased bacterial genera (*Prevotellaceae_UCG-004*, *Alloprevotella*, *Ruminococcaceae_UCG-011*, *Ruminococcaceae_UCG-005*, *Ruminococcaceae_UCG-004*, *Lachnospiraceae_UCG-010*, *Ruminiclostridium_5*, *Ruminococcus_2*, *Butyricicoccus*, *Coprococcus_3*, *Butyrivibrio_2*, *Fibrobacter* and *Lachnospiraceae_XPB1014_group*) were considered as intestinal beneficial bacteria, which play crucial roles in improving the digestion, metabolism, immunity and gut microbiota. *Ruminococcaceae*, a potential beneficial bacteria commonly found in colon and caecum, has been demonstrated to be involved in degrading cellulose and actively regulating intestinal environment and immunity [47]. Moreover, increased intestinal permeability, non-alcoholic fatty liver, and liver cirrhosis have all been linked to decreased *Ruminococcaceae* [48, 49]. Previous studies indicated that *Ruminiclostridium* residing in the intestine was conducive to increase the growth performance and decrease the gastrointestinal diseases [50]. *Ruminococcus* that mainly inhabit the rumen and hindgut showed the characteristics of degrading cellulose and starch [51]. The higher abundances of *Butyricicoccus* and *Lachnospiraceae* in the gut bacterial community is beneficial to alleviate intestinal inflammation [52, 53]. *Prevotellaceae* displayed the ability to degrade hemicellulose and high carbohydrate [54]. *Alloprevotella* was previously reported to reduce the risk of cardiovascular disease [55, 56]. As a butyrate-producing bacteria, *Butyrivibrio* not only decompose polysaccharides, cellulose and starch but also reduce the obesity-induced diabetes and cardiovascular dysfunctions through brain-gut axis [57, 58]. *Fibrobacter* can degrade cellulose [59]. Notably, the above-mentioned bacterial genera such as *Ruminococcaceae_UCG-011*, *Coprococcus_3*, *Ruminococcus_2*, *Ruminococcaceae_UCG-005*, *Ruminococcaceae_UCG-004*, *Butyrivibrio_2* and *Ruminiclostridium_5* were potential producers of short-chain fatty acids (SCFAs) in the intestine. SCFAs not only improve host immunity but also inhibit the colonization of pathogenic bacteria by regulating the pH of the intestines [60]. Moreover, SCFAs also play important roles in maintaining intestinal function and gut microbial balance [61]. Recent studies on the SCFAs also revealed their important role in anti-inflammatory, anti-cancer aspects and regulating energy intake. *Eubacterium ventriosum* can cause bacteremia and endocarditis.

Similar to the intestinal bacteria, intestinal fungi are also an important part of the gut microbiota, which play vital roles in intestinal ecosystem and host health [40]. Intestinal fungi have been demonstrated to induce intestinal inflammation and increase intestinal mucosal permeability [62]. Moreover, Li et al. indicated that gut fungal community was the promoter and participant of diarrhea in giraffe [35]. However, the gut fungal role and importance in yaks were chronically neglected due to their lower abundance. In this study, we dissected the shifts of the gut fungal community in yaks during diarrhea, which contribute to providing an insight into gut fungal community in yaks. Similar to the altered gut bacterial community, we observed that the taxonomic composition of gut fungal community changed significantly during diarrhea, characterized by an increase in the abundance of 3 fungal genera and a decline in 46 fungal genera. Among decreased fungi, some of them (*Neocallimastix*, *Periconia*, *Tilletiopsis* and *Mortierella*) were closely related to host digestion and health. The *Neocallimastix* can degrade polysaccharide and cellulose [63]. The metabolite of *Periconia* have antibacterial activity [64]. *Tilletiopsis* can produce antifungal compounds [65]. *Mortierella* can produce arachidonic acid [66]. Arachidonic acid plays important roles in the prevention of cardiovascular diseases, diabetes and tumors [67]. Moreover, some fungal genera (*Humicola*, *Mucor*, *Ramularia* and *Zoellneria*) in the gut fungal community of diarrheic yaks even cannot be detected, suggesting that their growth was significantly restricted.

Gut microbial dysbiosis has been deemed as the pathological mediators of many diseases. Under normal physiological conditions, these microbes inhabiting the gut can engage in a commensal, synergetic or antagonistic relationship, maintaining intestinal homeostasis [32]. Therefore, some gut bacterial and fungal alternations can affect the functions of other bacteria and fungi through interactions between microorganisms. Correlation network analysis of this study revealed a significant correlation between some dramatically altered bacteria and fungi affected by diarrhea, which may further affect overall intestinal functions. Moreover, these changed bacteria or fungi can also affect some bacteria or fungi that do not significantly change during diarrhea through the interaction between microorganisms, thereby expanding the influence of diarrhea on the gut microbiota and intestinal functions. These results indicated that diarrhea not only directly altered the gut bacterial and fungal compositions and diversities but also indirectly affected some bacteria and fungi through the interaction of microorganisms, which may further destroy the intestinal homeostasis and induce intestinal dysfunction.

## Conclusion

Taken together, this study dissected the shifts of gut bacterial and fungal communities in diarrheic yaks. Results revealed that the gut bacterial and fungal diversities of diarrheic yak were significantly decreased and accompanied by significant changes in taxonomic composition. This study also extended the understanding of gut bacterial and fungal characteristics in yaks with different health states and released a crucial message that the gut bacterial and fungal dysbiosis may be one of the causes of diarrhea in yaks. Furthermore, the present study may provide a theoretical basis for establishing diarrhea control system in yaks from the gut microbial perspective.

## Supplementary Information

**Additional file 1: Fig. S1.** Clustered heatmap of yaks in different health status at the bacterial genus level. The color values of the heatmap indicate the normalized relative richness of each species. **Fig. S2.** Clustered heatmap of yaks in different health status at the fungal genus level. The color values of the heatmap indicate the normalized relative richness of each species. **Fig. S3.** The gut bacterial comparisons between healthy and diarrheic yaks in phylum and genus levels. Metastats analysis was applied to identify the significantly differentially abundant bacterial genera between both groups and all of the data represent means ± SD. * p < 0.05, ** p < 0.01. **Fig. S4.** The gut fungal comparisons between healthy and diarrheic yaks in phylum and genus levels. Metastats analysis was applied to identify the significantly differentially abundant fungal genera between both groups and all of the data represent means ± SD. * p < 0.05, ** p < 0.01.

## Data Availability

Yes.
