# Supplementary material for: Comparative analysis of gut microbiota in healthy and diarrheic yaks
Source: Microb Cell Fact. 2022 Jun 3;21:111. doi: 10.1186/s12934-022-01836-y (PMC9164553; doi:10.1186/s12934-022-01836-y)
Supplement: Supplementary file 1 — Additional file 1: Fig. S1. Clustered heatmap of yaks in different health status at the bacterial genus level. The color values of the heatmap indicate the normalized relative richness of each species. Fig. S2. Clustered heatmap of yaks in different health status at the fungal genus level. The color values of the heatmap indicate the normalized relative richness of each species. Fig. S3. The gut bacterial comparisons between healthy and diarrheic yaks in phylum and genus levels. Metastats analysis was applied to identify the significantly differentially abundant bacterial genera between both groups and all of the data represent means ± SD. * p < 0.05, ** p < 0.01. Fig. S4. The gut fungal comparisons between healthy and diarrheic yaks in phylum and genus levels. Metastats analysis was applied to identify the significantly differentially abundant fungal genera between both groups and all of the data represent means ± SD. * p < 0.05, ** p < 0.01. [file 12934_2022_1836_MOESM1_ESM.docx]

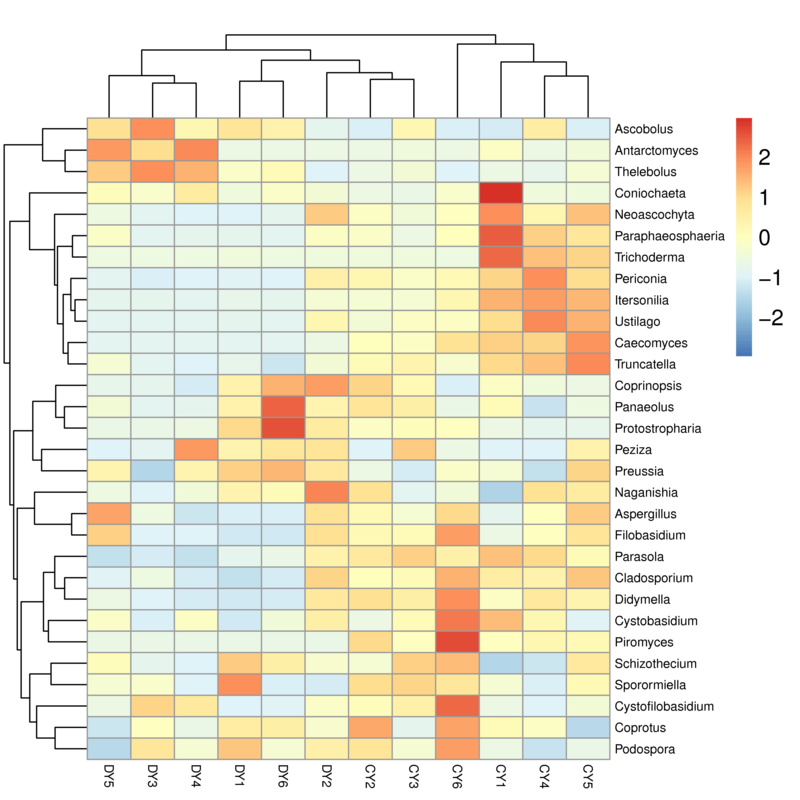


**Supplementary Fig. S1.** Clustered heatmap of yaks in different health status at the bacterial genus level. The color values of the heatmap indicate the normalized relative richness of each species.


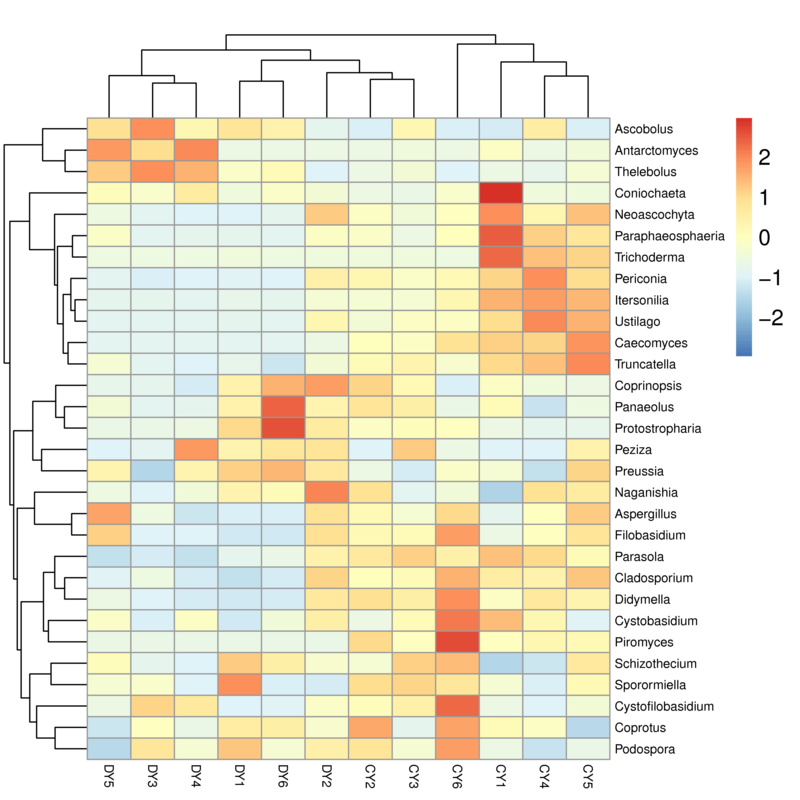


**Supplementary Fig. S2.** Clustered heatmap of yaks in different health status at the fungal genus level. The color values of the heatmap indicate the normalized relative richness of each species.


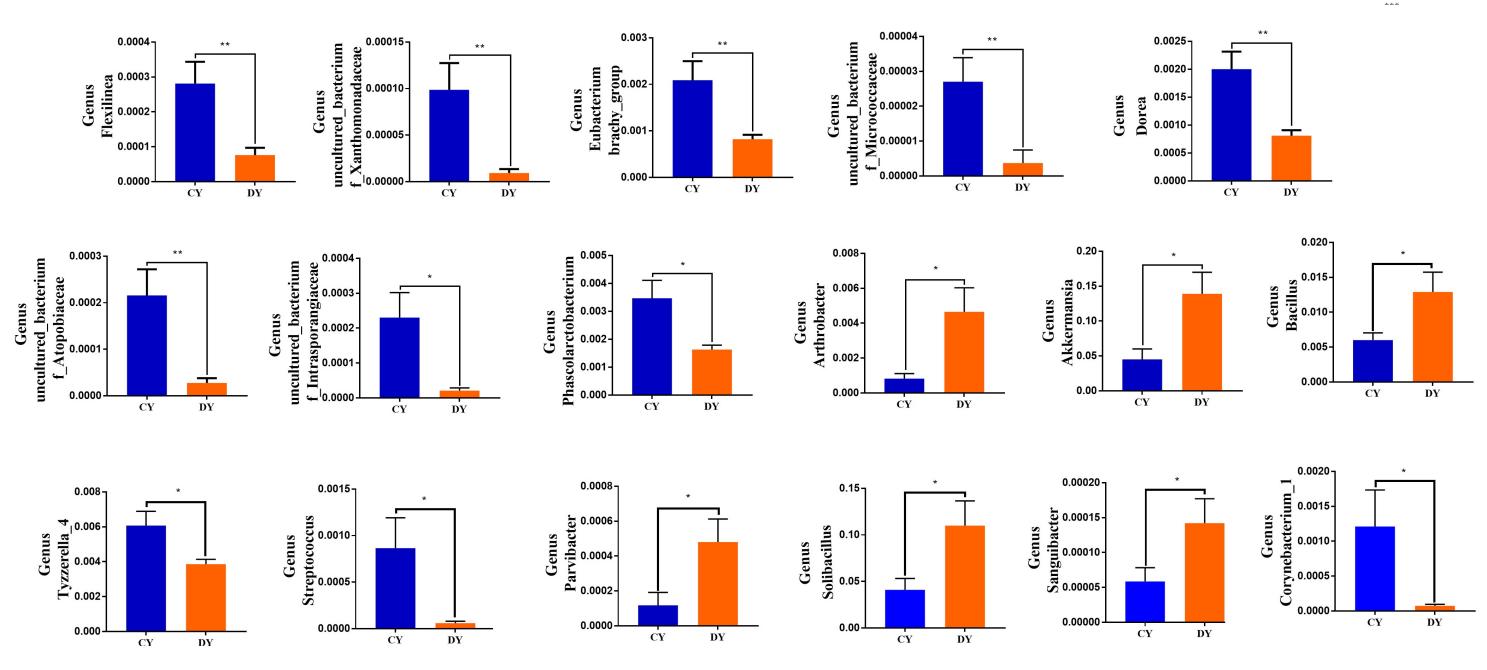


**Supplementary Fig. S3.** The gut bacterial comparisons between healthy and diarrheic yaks in phylum and genus levels. Metastats analysis was applied to identify the significantly differentially abundant bacterial genera between both groups and all of the data represent means ± SD. * p < 0.05, ** p < 0.01.


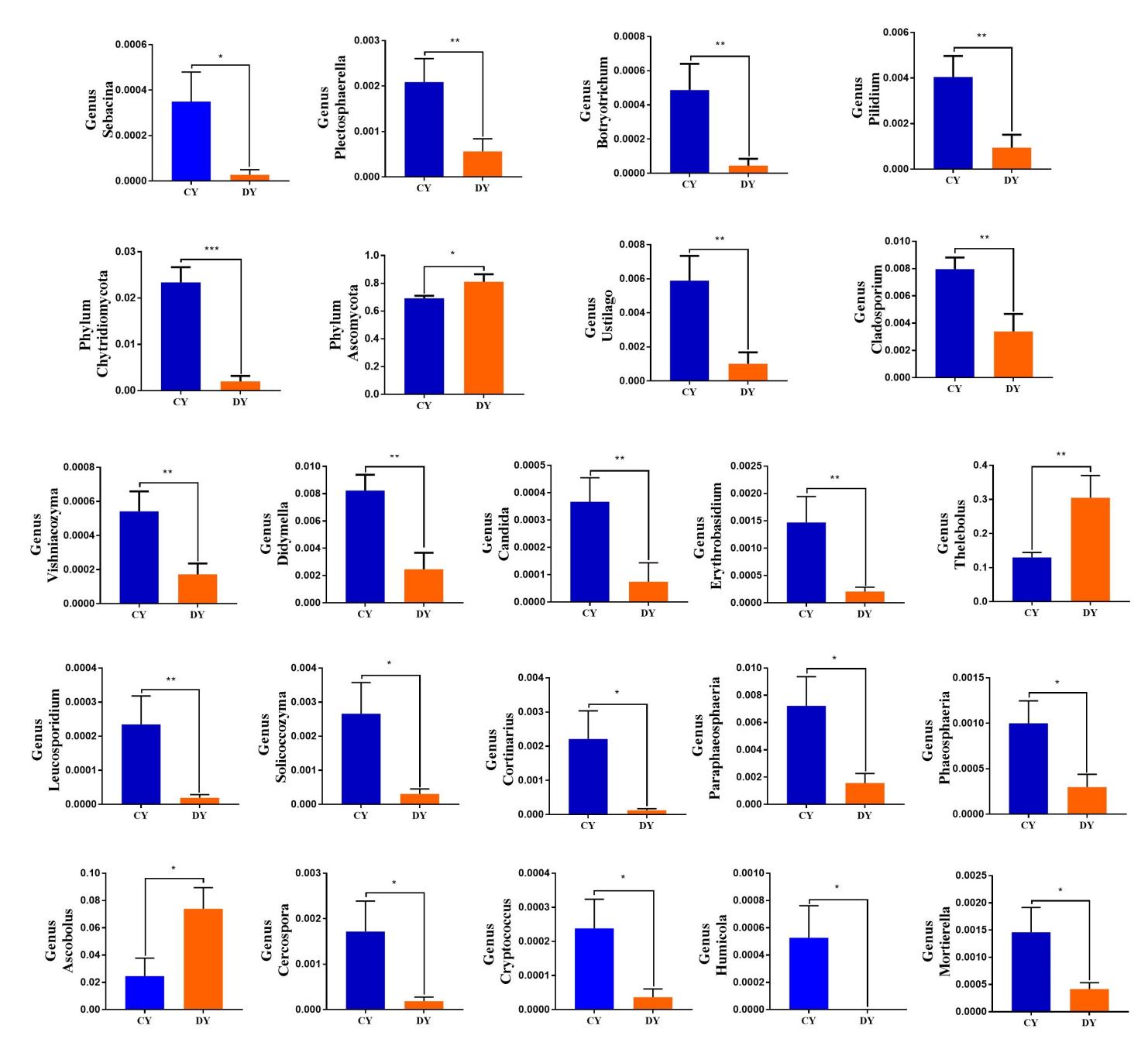


**Supplementary Fig. S4.** The gut fungal comparisons between healthy and diarrheic yaks in phylum and genus levels. Metastats analysis was applied to identify the significantly differentially abundant fungal genera between both groups and all of the data represent means ± SD. * p < 0.05, ** p < 0.01.
